# Food-Related Behavioral Patterns in Patients with Inflammatory Bowel Diseases: The Role of Food Involvement and Health Engagement

**DOI:** 10.3390/nu16081185

**Published:** 2024-04-16

**Authors:** Lorenzo Palamenghi, Dilara Usta, Salvo Leone, Guendalina Graffigna

**Affiliations:** 1 EngageMinds HUB—Consumer, Food & Health Engagement Research Center, Faculty of Agriculture, Food and Environmental Sciences, Catholic University of Sacred Heart, 26100 Cremona, Italy; lorenzo.palamenghi@gmail.com (L.P.); guendalina.graffigna@unicatt.it (G.G.); 2 National Association for Chronic Inflammatory Bowel Diseases (AMICI ETS), 20125 Milan, Italy; salvo.leone@amiciitalia.net; 3 Faculty of Psychology, Catholic University of Sacred Heart, 20123 Milan, Italy

**Keywords:** inflammatory bowel disease, emotional regulation, food choices, food involvement, patient engagement, cluster analysis

## Abstract

Nutrition has been acknowledged as crucial in IBD and is relevant to patients’ motives behind food choices, which are affected by health engagement (HE) and food involvement (FI). This study aimed to profile IBD patients according to their levels of health engagement and food involvement to identify patterns of different motives behind food choices, particularly regarding the use of food to regulate mood. A cross-sectional study was conducted with 890 Italian IBD patients who completed an online survey in April 2021. We measured health engagement, food involvement, motives behind food choices, emotional states, and food-related quality of life (Fr-QoL). K-means cluster analysis was performed to identify participants with similar levels of health engagement and food involvement. Four clusters were identified: “Health-conscious (high HE, low FI)”, “Balanced (high HE, high FI)”, “Hedonist (high FI, low HE)”, and “Careless (low FI, low HE)”. Clusters with high FI are inclined toward seeking pleasurable food, but when supported with high health engagement, individuals were less prone to use food to manage mood. Groups with higher health engagement demonstrated lower hospitalization rates and relapses and better Fr-QoL. Profiling IBD patients regarding FI and HE could aid clinicians in identifying individuals at greater risk of maladaptive food-related behaviors.

## 1. Introduction

Inflammatory bowel disease (IBD), encompassing Crohn’s disease (CD) and ulcerative colitis (UC), is a lifelong incurable condition, affecting approximately 0.2% of the European population and around five million worldwide, and represents a substantial and escalating global health challenge [1,2]. IBD manifests with several gastrointestinal symptoms characterized by alternating periods of exacerbation and remission [3], typically emerging as pain, diarrhea, abdominal discomfort, fatigue, reduced appetite, and weight loss, impacting daily living activities and resulting in a significant decrease in health-related quality of life [4]. These symptoms are initiated and triggered by environmental factors, such as diet, in individuals genetically susceptible to the condition, characterized by gut dysbiosis and abnormal immune response [5].

Extensive research demonstrated the impact of diet on IBD onset and prognosis [6,7], and the interplay between nutrition and gut microbiota was acknowledged as a significant factor in the pathogenesis of IBD [8]. To illustrate, the westernized lifestyle has been recognized as a critical contributor to the rising incidence of IBD and exacerbating intestinal symptoms [9,10], supported by the evidence of the effect of saturated fat, cholesterol, and food additive intake on gut inflammation [11]. Also, high protein intake, including red meat and processed meat, is significantly related to the increased risk of IBD development [12] and triggered IBD symptoms [9]. On the contrary, the Mediterranean diet is recognized as a healthy dietary regimen with anti-inflammatory effects and has been linked to a remarkably reduced risk of later onset CD [13] and improvement in inflammation and disease activity [14]. This captivating evidence underscores the significance of diet and nutrition as a regulatory factor in gut inflammation [7].

The scientific community has recognized the significance of dietary behaviors and nutrition in managing IBD [15]. Numerous studies have attempted to establish nutritional guidelines to clarify the impact of diet on the onset and progression of IBD [10,16]. However, evidence indicates a suboptimal adherence of IBD patients to recommendations [17]. So far, various barriers to adapting to a healthy diet have been introduced, such as a lack of knowledge in identifying foods, difficulties correlating disease symptoms with diet, obstacles to accessing IBD-friendly food, challenges with the complexity of diet, and changing habits and lifestyles [18]. Further, adherence to nutritional guidelines and healthy food choices highlights the pivotal role of psychological dynamics in the complex evaluations underpinning dietary decisions [19,20]. Evidence suggests that nutritional restrictions substantially impact psychological well-being by causing distress and negative mood in patients [21]. Frustration, distress, and feelings of deviating from normalcy may contribute to dysregulated food behaviors aimed at alleviating negative emotional states [22]. These coping mechanisms may remarkably influence dietary choices, with certain foods sought for comfort during distress or negative moods, establishing a link between emotional well-being and consumption patterns [23]. The regulatory role of food on mood is also supported by evidence pointing to psychological, hedonistic, and neurochemical pathways that facilitate emotional eating [24,25].

Building on this understanding, food involvement (FI) emerges as a significant psychological construct that interlinks with food utilization as a regulatory mechanism for mood [26]. Food involvement, described as the level of cognitive, behavioral, and emotional commitment a person gives to every aspect of food interaction, has been linked to various dietary patterns [27,28,29], suggesting that individuals who deeply engage with food, making it a central aspect of their lives, may be more susceptible to the psychological and behavioral tendencies discussed previously. Specifically, the renunciation of food could burden these individuals by exacerbating distress and complicating adherence to dietary guidelines [30]. This is particularly relevant considering their propensity for using food to regulate mood and their preference for hedonic, pleasure-inducing foods [31]. Thus, understanding the nuances of food involvement could illuminate pathways to mitigate the emotional distress associated with dietary restrictions, especially in populations with IBD.

Moreover, the complex relationship between psychological factors underscores the critical need for enhanced patient involvement in managing IBD. In this context, the health engagement (HE) concept appears to be a protective factor in adhering to nutritional guidelines and healthy food choices, elaborating on individuals’ emotional and value-based readiness to actively manage their health and lifestyle [32]. Health engagement entails a spectrum of behavioral skills empowering patients to self-manage their health condition, complemented by the requisite knowledge and self-efficacy to implement proactive behaviors [33,34]. Remarkably, health engagement within chronic care contexts has been linked to improved quality of life [33], enhanced health literacy [35,36], and greater adherence to behavioral prescriptions [37].

Unraveling the interplay between food involvement and health engagement could address clinicians’ daily practical concerns, including symptom management, monitoring disease progression, planning personalized interventions, providing nutritional guidance, offering psychosocial support, and empowering patients to improve the well-being of individuals managing IBD. Given the limited focus on psychological factors influencing dietary choices among the IBD population and the significant impact of diet on their well-being and symptom management [21,38], there is a need to understand the various factors driving their food choices, including reasons for consuming foods perceived as triggers. Through this study, we aim to uncover the relationship between food involvement and health engagement and assess their utility in delineating distinct groups of IBD patients with varying motives for food choices, particularly regarding the use of food to regulate mood. Furthermore, our study explores whether these identified patient groups exhibit differing food-related quality-of-life levels.

## 2. Materials and Methods

### 2.1. Study Design and Participants

A cross-sectional study was carried out between the 3 and the 19 of April 2021 as an online survey on the SurveyMonkey platform. Individuals older than 18 years old with a formal diagnosis of IBD were contacted by sending invitation emails through an Italian IBD patient organization, “AMICI ETS”, with a purposive sampling method and completed a survey. Individuals were ineligible to participate if they were unable to give informed consent and had impaired capacity to complete the survey. No incentives were given to the participants. Ethical approval was granted by the Ethics Committee of the Department of Psychology of the Università Cattolica del Sacro Cuore, Italy (protocol code: 36-21). All participants provided written informed consent.

### 2.2. Measures

The online survey comprised validated self-report questionnaires and ad hoc items. The instruments used in this study can be classified into two groups: (i) for clustering variables, including health engagement and food involvement, and (ii) for dependent variables, involving sociodemographic and disease-related characteristics, food choice motives, emotional states, and food-related quality of life.

#### 2.2.1. Sociodemographic and Disease-Related Characteristics

Sociodemographic and disease-related data included gender, age, diagnosis (Colitis, either ulcerative or indeterminate, or Crohn’s disease), duration of diagnosis, level of education (middle school or below, high school, university degree), existing comorbidities, whether had relapses or hospitalizations in the last year, whether had undergone surgery for IBD, and whether currently taking medications for IBD.

#### 2.2.2. Food Involvement

Participants’ food involvement was assessed using the Food Involvement Scale (FIS) [27], which addresses the perceived level of importance that a person attributes to food. The FIS consists of 12 items with a 7-point Likert score (1: strongly disagree, 7: strongly agree) regarding food acquisition, preparation, cooking, eating, and disposal and incorporates two dimensions: “set and disposal” (3 items) and “preparation and items” (9 items). An example of an item is “I don’t think much about food each day”. Total scores ranged between 12 and 84, with higher scores indicating higher food involvement.

#### 2.2.3. Health Engagement

Health engagement was assessed with the Patient Health Engagement Scale (PHE-s^®^), which was validated among various chronic conditions [32]. The scale is based on the Patient Health Engagement Model, a developmental psychological theory describing the patients’ experience of becoming active players in their healthcare pathway. The PHE-s^®^ can grasp the complex psychological experience of health engagement and has an ordinal structure consistent with the PHE model’s conceptualization, which, through an algorithm that provides the final score, envisages four different positions along the engagement continuum: Blackout, Alert, Adhesion, and Eudaimonic Project. An example of an item is “When I think about my illness, I feel overwhelmed by emotions”. This ordinal scale is measured on a 7-point scoring system to facilitate patient responses and avoid social desirability bias [32].

#### 2.2.4. Food Choice Motives

Food choice motives were evaluated with the revised single-item Food Choice Questionnaire (FCQ) [39]. The FCQ explores people’s motivations behind food choices by asking participants to rate each “motive” on a 7-point Likert scale (1: not at all important, 7: very important); it starts with the statement “It is important to me that the food I eat on a typical day is…” and with the following answer options: health, mood, convenience, taste, natural, price, weight control, familiar, environment, animal welfare, and social justice. One additional single-item construct was added to the scale, questioning how important it is for patients that food helps them manage their symptoms.

#### 2.2.5. Emotional States

On a scale from 0 to 100, participants were asked to rate how much they felt three positive emotional states—happiness, hopefulness, and satisfaction—and seven negative emotional states—sadness, fear, anger, disgust, anxiety, distress, and boredom—over the previous 24 h.

#### 2.2.6. Food-Related Quality of Life

Food-related quality of life was measured with the Food-Related Quality of Life Questionnaire (FR-QoL-29), a one-factor self-report measure developed based on qualitative interviews with the IBD population. The FR-QoL, which has good validity and reliability across various characteristics, was used to evaluate participants’ food-related quality of life, which refers to their relationship with food and how this impacts their daily lives and psychological well-being [40]. The FR-QoL-29 involves 29 items on a 5-point Likert scale (1: definitely agree, 5: definitely disagree), including four items reversed for scoring. An example of an item is “In the past two weeks, I have regretted eating and drinking things that have made my IBD symptoms worse”. Total scores ranged between 29 and 145, with higher scores denoting greater food-related quality of life.

### 2.3. Statistical Analysis

Statistical analyses were conducted using IBM^®^ SPSS 27 (IBM Corp., Armonk, NY, USA). Descriptive statistics of the sample were computed to assess the composition of the sample. Scales were scored according to the literature. Regarding the PHE-s^®^, a distinct methodology was employed due to limitations posed by its conventional scoring procedure [32], which yields an ordinal score spanning from 1 to 4 based on its theoretical framework [34], rendering it incompatible with the intended cluster analysis (please refer to the subsequent section). Consequently, the 7-point ordinal scales were re-encoded into 4 points to align with the standard scoring protocol, followed by applying a partial credit Rasch model (PCM). The PCM served the dual purpose of assessing the unidimensionality and fit of each ordinal item within the targeted construct while determining the score for each participant [41]. Specifically, the fit mean square (MNSQ) statistics (infit and outfit) were computed to evaluate adherence to the anticipated model; ideally, these statistics should fall within the range of 0.6 to 1.4 to signify a good fit with the Rasch model [42]. Furthermore, analyses of difficulty and step parameters were conducted to ensure adequate discrimination among the various response categories and to maintain the monotonic order.

To identify groups of participants with similar levels of patient health engagement and food involvement, a k-means cluster analysis was conducted utilizing the scores from the PHE-s^®^ and FIS as segmentation variables. Before the k-means clustering, the scores were standardized into z-scores. Outliers, defined as participants with either PHE-s^®^ or FIS z-scores exceeding |3|, were systematically excluded from the dataset. Determining the optimal number of clusters was facilitated through a progressive series of analyses, commencing with two clusters and iteratively increasing. Criteria employed to ascertain the most suitable cluster count encompassed the following:

(i) Interpretability of the final clusters’ averages and ANOVA’s *p*-values;
(ii) Number of participants in each cluster (closer to homogeneity is better);
(iii) Pseudo-F values, calculated according to the procedure described by Calinski and Harabasz [43]: higher pseudo-Fs are an indication of a better solution;
(iv) Finally, to address the stability of the identified best solution, the Rand index [44] was calculated; the Rand index is considered acceptable above the 0.70 threshold.

A combination of Pearson’s χ^2^ tests and one-way Welch’s ANOVAs was employed to evaluate disparities across the identified clusters concerning the aforementioned dependent variables (as detailed in Section 2.2). Significant χ^2^ tests were followed by an inspection of adjusted standardized residuals, where significance was attributed to values exceeding |2| [45]. Additionally, Cramér’s V was computed as an indicator of effect size. Games–Howell post hoc tests followed significant ANOVAs. The effect size was quantified using η^2^. For variables violating the assumption of normality in the ANOVA (skewness and/or kurtosis > |1|), the robustness of findings was assessed by re-running the model post a logarithmic transformation, followed by reassessment of normality parameters. However, for interpretability, results are primarily reported based on non-transformed variables unless otherwise indicated, owing to discrepancies observed between outcomes derived from transformed and non-transformed variables. A *p*-value of <0.05 was considered statistically significant.

## 3. Results

### 3.1. Participant Characteristics

A total of *n* = 1113 participants responded to the survey. Among them, *n* = 211 were excluded due to incomplete responses and the premature termination of the online survey. Additionally, *n* = 12 participants were deemed outliers; consequently, the final analysis included data from *n* = 890 patients. Table 1 provides an overview of participants’ characteristics. The average age of the participants was 47 ± 14 years (min 18, max 85), and on average, patients reported receiving their diagnosis 17 ± 11.5 years ago (min 0.5, max 51).

### 3.2. Scale Scoring and Descriptive Statistics

The analysis of the difficulty and the step parameters from the partial credit Rasch model applied to the PHE-s^®^ items indicated a satisfactory ranking of the various response categories, with each scale item adhering to a monotonic order. Furthermore, the infit and outfit statistics fell within acceptable ranges, with values ranging from 0.75 to 0.82 and 0.70 to 0.82, respectively.

Table 2 demonstrates the descriptive statistics of the FIS and PHE-s^®^ before being transformed into z-scores. Both variables showed an acceptably normal distribution, and ordinal scores were calculated to assess the overall distribution of PHE-s^®^ phases by the underlying theoretical framework (Patient Health Engagement Model) [34]. The findings indicate that most participants were in either the Adhesion phase (*n* = 429), indicative of a moderate degree of health engagement, or the Arousal phase (*n* = 298). A higher level of health engagement, namely, the “Eudaimonic project”, was achieved by only 131 participants, while merely 32 individuals were categorized at the lowest conceivable level, denoted as “Blackout”.

Regarding the FCQ, the percentages of patients that answered “a lot” (5, 6, or 7 on the Likert scale) were also calculated for each item of the FCQ. Overall, “helps me control my symptoms” was responded to by most patients (78%), as well as “it’s healthy” (73%). Being pleasant (59%) and natural (58%) are also reported by most patients as essential drivers.

Remarkably, emotional states and food-related quality of life were correlated with some motives behind food choices and with PHE-s^®^. Specifically, findings from Spearman’s correlation analyses revealed that overall positive emotions were strongly and positively correlated with the PHE-s^®^ score (ρ = 0.597; *p* < 0.001) and demonstrated a strong, negative correlation with the Fr-QoL (ρ = −0.400; *p* < 0.001). Additionally, positive emotions were found to negatively correlate with the use of food as a means to regulate mood (ρ = −0.131; *p* < 0.001) and, intriguingly, with the management of symptoms (ρ = −0.119; *p* < 0.001). Finally, positive emotions also showed small correlations with using convenient, easy-to-prepare food (ρ = −0.096; *p* = 0.004) and attention to price (ρ = −0.072; *p* = 0.033).

On the other hand, negative emotions had a strong, negative correlation with PHE-s^®^ scores (ρ = −0.575; *p* < 0.001) and a strong, positive correlation with Fr-QoL-29 scores (ρ = −0.464; *p* < 0.001). Coherently with the results reported for positive emotions, the negative emotion score was positively correlated with the use of food as a means to regulate mood (ρ = 0.173; *p* < 0.001) and with the preference for convenient food (ρ = 0.118; *p* < 0.001). Interestingly, consistent with the described construct, the FIS did not correlate with emotional states but positively correlated with the Fr-QoL (ρ = 0.143; *p* < 0.001). Finally, a strong and anticipated correlation emerged between the PHE-s^®^ and the Fr-QoL-29 scores (ρ = −0.574; *p* < 0.001).

### 3.3. Cluster Analysis

Four distinct solutions (comprising two, three, four, and five clusters) were explored to categorize patients based on their PHE-s^®^ and FIS scores. Among these, the four-cluster solution emerged as the most optimal, characterized by clusters with interpretable means and a reasonably balanced distribution of participants. Additionally, Pseudo-F values were computed for each solution, with the four-cluster solution yielding the highest value (473.055, 567.422, 610.208, and 577.528 for the two, three, four, and five-cluster solutions, respectively). Furthermore, the Rand index for the four-cluster solution was calculated to be 0.94, surpassing the acceptability threshold. Table 3 presents the average scores of the final clusters and the outcomes of the ANOVA test, depicting the variations across clusters and the number of subjects in each cluster.

The four identified clusters exhibited distinct levels of both PHE-s^®^ and FIS scores (F3, 476.93 = 626.597; *p* < 0.001; η^2^ = 0.681; F3, 477.12 = 594.238; *p* < 0.001; η^2^ = 0.667, respectively). The identified clusters are as follows:

(i) A cluster comprising patients with higher PHE-s^®^ scores and lower FIS scores is labeled as the “Health-conscious” group.
(ii) Another group characterized by higher scores on PHE-s^®^ and FIS is the “Balanced” group.
(iii) A cluster with higher FIS scores and lower PHE-s^®^ scores is identified as the “Hedonist” group.
(iv) Lastly, a cluster of patients with lower scores on both PHE-s^®^ and FIS is called the “Careless” group.

#### 3.3.1. Differences between Clusters

##### Sociodemographic and Disease-Related Characteristics

Overall, the results indicate that the identified clusters are not strongly correlated with sociodemographic characteristics. Specifically, χ^2^ tests revealed a moderate association between belonging to a cluster and gender (χ^2^_(1, 890)_ = 39.529; *p* < 0.001; V = 0.21). Post hoc examination of adapted standardized residuals revealed that the “Health-conscious” cluster exhibited a higher percentage of males (z = 5.2), while the “Balanced” and “Hedonist” clusters had a higher rate of females (z = 2.5 and z = 4.3, respectively).

Furthermore, the results revealed a weak association between belonging to a cluster and recent hospitalizations (χ^2^_(1, 890)_ = 24.616; *p* < 0.001; V = 0.16) and a moderate association with recent relapses (χ^2^_(1, 890)_ = 92.681; *p* < 0.001; V = 0.32). Specifically, participants in the “Health-conscious” group exhibited less frequent recent hospitalizations and relapses (z = 4.1 and z = 7.0, respectively), while “Balanced” participants experienced fewer recent relapses (z = 3.1). Conversely, “Hedonists” were more likely to have recent hospitalizations and relapses (z = 3.8 and z = 6.9, respectively), and “Careless” participants showed a higher frequency of recent relapses (z = 4.2).

Finally, Welch’s ANOVA revealed a significant difference in age across clusters (F3, 476.92 = 13.991; *p* < 0.001; η^2^ = 0.045). Post hoc Games–Howell tests indicated that participants in the “Health-conscious” group had a higher average age (M = 51.07; SD = 14.29) compared to the other clusters (M = 46.76, SD = 12.54; M = 42.8, SD = 12.74; and M = 46.15, SD = 13.89, for clusters 2, 3, and 4, respectively). Additionally, the “Balanced” group had a higher mean age than the “Hedonist” group. However, the differences between Clusters 2 and 4 and Clusters 3 and 4 were not statistically significant. Table 4 presents the results of χ^2^ analyses and the percentages of the various group characteristics.

##### Food-Related Quality of Life

Regarding the food-related quality of life, the analysis revealed significant differences in Fr-QoL scores among participants in different clusters (F3, 482.70 = 104.161; *p* < 0.001; η^2^ = 0.257). Specifically, participants in the “Health-conscious” and “Balanced” groups had lower Fr-QoL scores (M = 2.66, SD = 0.79, and M = 2.81, SD = 0.76, respectively) compared to the other groups, indicating a higher quality of life related to food. On the other hand, “Careless” participants had higher scores than those in the “Health-conscious” and “Balanced” groups but lower scores compared to “Hedonists”, who exhibited the overall lowest food-related quality of life.

##### Motives behind Food Choices

Using the single items from the FCQ, the analysis revealed that participants in different clusters attributed varying degrees of importance to different motivations behind their food choices (F tests reported in Table 5 for each item). Specifically, participants in the “Balanced” and “Hedonist” clusters expressed greater concern for the “healthiness” of their food compared to those in the “Health-conscious” and “Careless” groups. Moreover, they exhibited a higher interest in food that provides pleasurable sensations. At the same time, participants in the “Health-conscious” cluster, along with the “Careless” group, showed the slightest interest in this aspect.

Individuals with high health engagement (i.e., “Health-conscious” and “Balanced” clusters) were less inclined to use food as a means to manage mood compared to those in the “Hedonist” and “Careless” clusters. Notably, the “Hedonist” cluster was particularly interested in using food to control symptoms, while “Health-conscious” participants showed the slightest interest. Furthermore, “Hedonists” expressed a higher interest in affordability than “Health-conscious” participants. At the same time, a marginally significant difference (*p* = 0.051) indicated that the “Balanced” group exhibited the most interest in environmental friendliness. Conversely, participants in the “Careless” group showed little interest in environmental friendliness. Table 6 summarizes the different identified characteristics of these four groups of patients.

## 4. Discussion

The results of the current study, derived from a comprehensive clustering analysis based on the level of low-high health engagement and food involvement, uncovered intriguing insights into the multifaceted nature of food choices among individuals living with IBD.

In particular, food involvement holds a significant influence on the dietary preferences of individuals with IBD, as evidenced by our findings, which resonate with the existing literature [46], particularly regarding the pivotal role of food involvement in influencing food preferences. Our participants had a moderate level of food involvement, and consistent with prior research [47], individuals with higher levels of food involvement showed a predisposition toward healthier food choices, which underscores the significance of food-related attitudes and interests in shaping dietary behaviors. This inclination toward healthier eating patterns may stem from heightened nutritional awareness and appreciation among individuals with elevated food involvement, as they prioritize foods that align with health goals and dietary guidelines. Moreover, the multifaceted nature of food involvement, encompassing cognitive, emotional, and sensory dimensions, may have shaped food preferences and consumption patterns in this population [48]. Despite the lack of association with emotional states, the positive correlation between the Fr-QoL and food involvement implies that while emotional experiences may not directly influence food involvement, individuals who are more engaged with food-related activities and decisions may perceive a greater sense of satisfaction or fulfillment in their overall quality of life, particularly in the context of dietary aspects. This finding highlights the multifaceted nature of quality of life in chronic illness, where factors such as food involvement play a distinct role in shaping individuals’ perceptions of well-being.

The current study elucidated associations between emotional states and health engagement among IBD patients. Contrary to Barello and colleagues’ findings [33], our participants had a moderate level of health engagement, indicating a noteworthy interest in health-related behaviors despite the challenges posed by their medical condition. Notably, a robust positive correlation between overall positive emotions and health engagement underscores the influence of emotional well-being on individuals’ inclination toward health-conscious behaviors [34,49]. This finding aligns with another study conducted on a diabetes population that has manifested the synergy among these subjective experiences in terms of sustainable and effective disease management [50]. Therefore, individuals experiencing more positive emotions may exhibit greater motivation and commitment toward actively engaging in health-promoting activities, including dietary choices, despite the challenges posed by their medical condition.

Further exploration into the motivations underlying food choices reveals a complex interplay between nutritional considerations and emotional elaboration. The identification of four distinct patient clusters—“Health-conscious (high HE, low FI)”, “Balanced (high HE, high FI)”, “Hedonist (high FI, low HE)”, and “Careless (low FI, low HE)”—led to a nuanced understanding of the underlying motivations and behavioral patterns guiding dietary decisions. Regarding the individuals with high food involvement, an interesting difference emerged: “Hedonist” patients attributed higher importance to the capacity of food to help regulate mood and alleviate distress compared to “Balanced” patients. Indeed, individuals with high food involvement are inclined toward seeking pleasurable food [51]; however, supported by high health engagement, individuals demonstrated improved symptomatology and emotional well-being, thereby reducing the necessity to resort to food as a mechanism for emotional regulation [21]. Recent research by Wardle et al. (2018) revealed that low mood and high anxiety in patients with Crohn’s disease were associated with more frequent binge eating and decreased control of food cravings [52]. We claim that when individuals with high food involvement lack positive adaptation to their condition and experience worsened emotional states, they may become more prone to abuse comfort food as a means of mood regulation. Furthermore, it is interesting that the “Health-conscious” group appears to lack a prevalent driver for food selection compared to the other three clusters. This phenomenon could be attributed to the superior clinical status of these patients in comparison to the two groups demonstrating low health engagement.

While the four clusters did not represent particular differences regarding their sociodemographic characteristics (except the gender distribution), the groups with high health engagement levels demonstrated lower hospitalization rates and relapses. A recent study reported that health-engaged individuals are more likely to adhere to their treatment plans, including medication regimens and lifestyle modifications, leading to better disease control and reducing the likelihood risk of hospitalizations [53]. Furthermore, our results revealed a significant positive correlation between health engagement and food-related quality of life, indicating that a higher health engagement level is associated with a better perception of food-related factors on the overall quality of life among IBD patients. This implies that individuals actively engaged in managing their health may perceive fewer limitations or disruptions in their daily lives stemming from food-related issues and tend to have improved psychological well-being, potentially due to better coping strategies or adaptive behaviors. Similarly, higher levels of quality of life have been linked with higher health engagement among individuals with IBD [33,54]. In addition, individuals with the lowest food-related quality of life levels were classified into the “Hedonist” cluster, and their high food involvement may have contributed to increased cravings. In this sense, these cravings may be exacerbated by dietary restrictions imposed upon individuals in the “Hedonist” group, thereby impacting their overall quality of life related to food.

### 4.1. Limitations

The current study is subject to some limitations. Initially, the k-means cluster analysis serves as an exploratory technique. While the derived solution comprising four clusters proved to be interpretable and stable, exhibiting favorable metrics such as the Rand index, it is imperative to acknowledge the potential existence of alternative solutions. Secondly, the influence of symptoms such as abdominal pain, diarrhea, and bloating may prompt individuals to modify their diets in an attempt to alleviate discomfort and manage their condition effectively. Then, all participants in this study were voluntarily recruited from a patient organization, potentially introducing bias into our sample and limiting the generalizability of findings to all patients with IBD. While the study was conducted in 2021, it is important to note that the fundamental principles underlying dietary behaviors and health management strategies are unlikely to undergo significant changes over a relatively short period. Also, this investigation focused exclusively on Italian patients, whose unique food culture may hinder the reproducibility of results in populations where food holds different cultural significance. Finally, the instruments used in this study relied on self-reported data; however, it is worth mentioning that the instruments, apart from the measure of emotional state, are validated to reduce potential biases. The choice to use a scale from 0 to 100 for measuring emotional state in our study was deliberate and aligned with our research objectives; nonetheless, we aim to incorporate alternative measures in future research endeavors. To mitigate these limitations, future research endeavors should address these constraints and validate and extend these findings across diverse cultural contexts.

### 4.2. Relevance for Clinical Practice

By integrating strategies to mitigate the impact of emotional eating on symptom severity and disease progression, healthcare providers can optimize patient outcomes and enhance overall quality of life. For instance, patients with low health engagement coupled with high food involvement may benefit from interventions targeting two key objectives: firstly, enhancing their health engagement to foster sustained behavioral change and, secondly, assisting them in exploring alternative mood modulation strategies beyond reliance on food. Conversely, individuals characterized by high levels of both health engagement and food involvement necessitate interventions primarily focused on maintaining health engagement and cultivating a positive mindset rather than solely educational endeavors. Such patients have either initiated or are in the process of implementing positive dietary changes, which are not inherently at odds with their keen interest in food. Nevertheless, due to the inherent challenges associated with adhering to behavioral modifications, ongoing monitoring and support from healthcare professionals are essential to facilitate the maintenance of these changes.

From a clinical perspective, our findings highlight the need for personalized and holistic approaches to dietary management in IBD patients. Furthermore, implementing psychological profiling for patients could facilitate the derivation of robust inferences regarding their motivational styles and the underlying motivations for their dietary selections. Developing approaches to education and adherence support specifically tailored to cater to the distinct motivational dimensions characteristic of particular profiles makes it feasible to leverage these aspects strategically. Such differentiated, bespoke approaches should be co-designed considering the psychological and motivational profiles of diverse patients. This strategy may promote a more patient-centered approach to engaging individuals in self-management and adherence practices, enhancing the likelihood of achieving sustained and positive behavioral change.

## 5. Conclusions

In conclusion, our study offers valuable insights into the intricate motivations guiding food choices among individuals with IBD. Based on our findings, food involvement significantly impacted the dietary preferences of the participants, with higher levels of food involvement associated with the inclination to seek pleasurable food. However, individuals with high health engagement levels reflected a better emotional state which resulted in less need for food to regulate their mood. Despite the lack of direct association with emotional states, individuals highly engaged with health experienced a greater sense of satisfaction and fulfillment in their overall quality of life, particularly concerning dietary aspects. This might be due to the fact that patients with a higher PHE-s^®^ score have less severe symptomatology and thus, potentially, fewer problems with food. Moreover, given the importance of understanding patients’ subjective experiences, utilizing measurement tools could be useful to identify patterns in the dietary habits of IBD patients and design personalized interventions to promote disease management. Future research exploring the relationship between psychological-behavioral variables and clinical outcomes is strongly recommended.

## Figures and Tables

**Table 1:** Participants’ sociodemographic and disease-related characteristics (*n* = 890).

Participant Characteristics	*n*	%
Gender		
Male	355	39.9
Female	535	60.1
Diagnosis		
Colitis (ulcerative or indeterminate)	445	50.0
Crohn’s disease	445	50.0
Level of education		
Middle school or lower	87	9.8
High school	422	47.4
University or higher	381	42.8
Existing comorbidities		
Yes	197	22.1
No	693	77.9
IBD relapses/hospitalizations in the last year		
Yes	352	39.6
No	538	60.4
Ever undergone surgery for IBD		
Yes	336	37.8
No	554	62.2
Currently taking drugs/medications for IBD		
Yes	784	88.1
No	106	11.9

**Table 2:** Descriptive statistics for the scales and single items.

Variables	Min	Max	Mean	SD	Skewness	Kurtosis
Food-related quality of life (Fr-QoL-29)	1.00	4.97	3.10	0.85	−0.344	−0.443
Food choice motives (FCQ) (It is important to me that the food I eat on a typical day…)						
is healthy	1	7	5.96	1.02	−1.275	2.645
is a way of monitoring my mood	1	7	5.03	1.50	−0.810	0.329
is convenient	1	7	4.99	1.42	−0.835	0.568
provides me with pleasurable sensations	1	7	5.57	1.11	−1.105	1.987
is natural	1	7	5.58	1.18	−0.878	1.023
is affordable	1	7	4.93	1.39	−0.618	0.322
helps me control my weight	1	7	4.75	1.66	−0.696	−0.154
is familiar	1	7	4.47	1.41	−0.502	0.134
is environmentally friendly	1	7	5.28	1.29	−0.755	0.626
is animal friendly	1	7	5.18	1.42	−0.628	−0.027
is fairly traded	1	7	4.47	1.14	−0.410	0.038
helps me control my symptoms	1	7	6.18	0.96	−1.224	1.753
Emotional states						
Happiness	0	100	58.66	26.37	−0.571	−0.331
Sadness	0	100	36.46	27.68	0.514	−0.694
Hopefulness	0	100	54.93	29.13	−0.235	−0.865
Fear	0	100	28.72	27.95	0.868	−0.239
Satisfaction	0	100	52.49	27.60	−0.180	−0.860
Anger	0	100	33.49	30.43	0.670	−0.776
Disgust	0	100	19.44	25.92	1.56	1.550
Anxiety	0	100	37.37	30.19	0.423	−0.977
Distress	0	100	46.65	31.28	0.090	−1.170
Boredom	0	100	29.65	29.30	0.778	−0.524
Food involvement (FIS)	2.75	6.33	4.57	0.64	−0.76	−0.308
Health engagement (PHE-s^®^)	−5.52	8.28	2.40	2.86	−0.148	−0.337

Abbreviations: SD, standard deviation; PHE-s^®^, Patient Health Engagement Scale; FIS, Food Involvement Scale; Fr-QoL, Food-Related Quality of Life Questionnaire; FCQ, Food Choice Questionnaire.

**Table 3:** Distribution of participants in clusters and results from ANOVA.

Variables	Clusters				Welch’s F	*p*	η^2^
	1: Health-Conscious (*n* = 261)	2: Balanced (*n* = 241)	3: Hedonist (*n* = 187)	4: Careless (*n* = 201)			
PHE-s^®^ cluster mean	0.75 ^a^	0.71 ^a^	−0.90 ^b^	−0.94 ^b^	626.597	<0.001	0.681
FIS cluster mean	−0.80 ^c^	0.77 ^b^	0.92 ^a^	−0.73 ^c^	594.238	<0.001	0.667

Abbreviations: PHE-s^®^, Patient Health Engagement Scale; FIS, Food Involvement Scale. Note: *p*, significance level; η^2^, eta-squared; mean scores with the same superscript letter do not differ significantly (*p* > 0.05) from each other (Games–Howell post hoc test). Superscript letters are ordered from the highest value to the lowest.

**Table 4:** Distribution of sociodemographic and clinical characteristics across clusters.

Variables	Clusters				χ2	*p*	Cramer’s V
	1: Health-Conscious	2: Balanced	3: Hedonist	4: Careless			
Gender					39.529	<0.001	0.21
Male	**53.3% (5.2)**	33.2% (−2.5)	26.2% (−4.3)	43.3% (1.1)			
Female	46.7% (−5.2)	**66.8% (2.5)**	**73.8% (4.3)**	56.7% (−1.1)			
Diagnosis							
Colitis	48.7% (−0.5)	48.5% (−0.5)	47.1% (−0.9)	**56.2% (2.0)**			
Crohn’s disease	51.3% (0.5)	51.5% (0.5)	53.9% (0.9)	43.8% (−2.0)	4.418	n. s.	
Level of education					7.525	n. s.	-
Middle school or lower	11.5% (1.1)	8.7% (−0.6)	5.9% (−2.0)	12.4% (1.4)			
High school	47.9% (0.2)	46.9% (−0.2)	46.5% (−0.3)	48.3% (0.3)			
University or higher	40.6% (−0.9)	44.4% (0.6)	47.6% (1.5)	39.3% (−1.1)			
Comorbidities					0.132	n. s.	-
Yes	22.2% (0.0)	21.6% (−0.2)	23.0% (0.3)	21.9% (−0.1)			
No	77.8% (0.0)	78.4% (0.2)	77.0% (−0.3)	78.1% (0.1)			
Hospitalizations					24.616	<0.001	0.16
Yes	4.6% (−4.1)	10.8% (−0.4)	**19.3% (3.8)**	13.9% (1.2)			
No	**95.4% (4.1)**	89.2% (0.4)	80.7% (−3.8)	86.1% (−1.2)			
Relapses					92.681	<0.001	0.32
Yes	21.8% (−7.0)	31.1% (−3.1)	**61.5% (6.9)**	**52.2% (4.2)**			
No	**78.2% (7.0)**	**68.9% (3.1)**	38.5% (−6.9)	47.8% (−4.2)			

Note: *p*, significance level; χ^2^, chi-square; n. s., not significant; Cramer’s V, effect size; cell values represent the percentages of the relative characteristic (e.g., male) in each cluster. Values in brackets represent standardized residuals, and cells highlighted in bold indicate where residuals show a significantly higher percentage relative to the sample at 5% significance (standardized residuals ≥ 2).

**Table 5:** ANOVA results regarding food-related quality of life and food choice motives.

Variables	Clusters				Welch’s F	*p*	η^2^
	1: Health-Conscious	2: Balanced	3: Hedonist	4: Careless			
Food-related quality of life	2.66 ^c^	2.81 ^c^	3.68 ^a^	3.49 ^b^	104.161	<0.001	0.257
Food choice motives (It is important to me that the food I eat on a typical day…)							
is healthy	5.84 ^b^	6.16 ^a^	6.02 ^a^	5.81 ^b^	6.749	<0.001	0.020
is a way of monitoring my mood	4.64	4.98 ^b^	5.42 ^a^	5.24	12.476	<0.001	0.038
is convenient	-	-	-	-	1.776	n. s.	-
provides me with pleasurable sensations	5.38 ^b^	5.70	5.73 ^a^	5.52 ^a,b^	5.193	0.002	0.017
is natural	-	-	-	-	2.344	n. s.	-
is affordable	4.75 ^b^	5.03 ^a,b^	5.13 ^a^	4.87 ^a,b^	3.668	0.012	0.011
helps me control my weight	-	-	-	-	0.636	n. s.	-
is familiar	-	-	-	-	0.626	n. s.	-
is environmentally friendly	5.33 ^a,b^	5.40 ^a^	5.28 ^a,b^	5.05 ^b^	2.604	0.051	0.010
is animal friendly	-	-	-	-	1.013	n. s.	-
is fairly traded	-	-	-	-	1.854	n. s.	-
helps me control my symptoms	5.90 ^c^	6.18 ^b^	6.43 ^a^	6.31 ^a,b^	13.054	<0.001	0.044

Note: *p*, significance level; η^2^, eta-squared; n. s., not significant, mean scores with the same superscript letter do not differ significantly (*p* > 0.05) from each other (Games–Howell post hoc test). Superscript letters are ordered from the highest value to the lowest.

**Table 6:** Result summary and principal characteristics of participants in each cluster for type of variable.

Variables	Clusters			
	1: Health-Conscious	2: Balanced	3: Hedonist	4: Careless
Defining variables	High health engagement and low food involvement	High health engagement and high food involvement	Low health engagement and high food involvement	Low health engagement and low food involvement
Sociodemographic and clinical characteristics	Males, with no hospitalization or relapses in the last year, higher average age	Females, with no relapses in the last year	Females with relapses and/or hospitalizations in the previous year	Diagnosis of colitis, with relapses in the last year and no surgical treatment in the past
Food-related quality of life and emotional state	High food-related quality of life	High food-related quality of life	Reported food-related quality of life is the lowest among groups	Reported food-related quality of life is low
Food choice drivers	This group does not show a particular driver regarding food. Compared to the other groups, these participants reported less interest in healthiness, mood modulation, sensations, affordability, and friendliness toward the environment. Food is not deemed essential for symptom management either	More interest in food’s healthiness and its pleasurable sensations. However, seeking pleasure is unrelated to mood modulation, as they reported this driver as less critical. Attention toward the environment and symptom management are also present (the latter is not as high as for other clusters)	Higher interest in food’s healthiness and its pleasurable sensations. In this case, however, there is also a reported use of food as a means to regulate mood, which is an essential driver for this group. Affordability and symptom control are also important drivers	This group shows a low interest in the healthiness of food and a high use of food for mood regulation. Inconsistently, they also report the importance of managing symptoms through food. Environmental friendliness is not essential

## Data Availability

The data supporting this study’s findings are available on request from the corresponding author. The data are not publicly available due to privacy or ethical restrictions.
